# Response of adult dragonflies to artificial prey of different size and colour

**DOI:** 10.1371/journal.pone.0179483

**Published:** 2017-06-29

**Authors:** Tammy M. Duong, Ann B. Gomez, Thomas N. Sherratt

**Affiliations:** Ottawa-Carleton Institute of Biology, Department of Biology, Carleton University, Ottawa, Canada; University of Sussex, UNITED KINGDOM

## Abstract

Aposematism is an evolved, cross-species association between a preys’ unprofitability and the presence of conspicuous signals. Avian predators have been widely employed to understand the evolution of these warning signals However, insect predators are abundant, diverse, and highly visual foragers that have been shown to be capable of learned aversion. Therefore, it is likely that their behaviour also shapes the nature of anti-predator traits. In this study, we evaluated the rates of attack of a community (13 species) of mature adult dragonflies (Odonata) on artificial prey of varying size (2.5–31 mm lengthwise) and colour pattern (black, black/yellow striped). The relative attack rates of dragonflies on prey increased as prey size decreased, but there was no evidence that the attack rates by dragonflies were affected by prey colour pattern and no evidence for an interaction between colour pattern and size. To investigate prey selection by specific predator species under field conditions, we compared the time to attack distributions of black-painted prey presented to two common dragonflies: *Leucorrhinia intacta* and the larger, *Libellula pulchella*. We found that the two dragonfly species, as well as the two sexes, had different foraging responses. *L*. *pulchella* was more likely to attack larger prey, and females of both species more likely to attack prey than males. Collectively, our results indicate that dragonflies are highly size selective. However, while the nature of this selectivity varies among dragonfly species, there is little evidence that classic black/yellow warning signals deter attack by these aerial invertebrate predators.

## Introduction

Animal colouration is a key component of anti-predator defence, allowing organisms to match their backgrounds for camouflage [1], to startle predators [2] and warn potential predators that they are inedible or otherwise costly to attack [3]. This latter association between defence and conspicuous coloration (generally interpreted as a “warning signal” [4]) is known as “aposematism” [5]. Experiments on the cognition and behaviour of birds have long been used to help understand the initial evolution and maintenance of these warning signals [6,7]. Yet invertebrates–ranging from spiders to asilid flies–are widespread, diverse, voracious insectivores, and often outnumber birds in a given area by an order of magnitude. Moreover, many of these predators can discriminate among conspicuous signals and have been shown to be capable of aversion learning, a major component in the evolution of warning signals and mimicry [3]. For example, mantids can learn to differentiate harmless and noxious (shock-delivering) blowflies depending on the colour of background they were presented on [8]. Moreover, after learning to avoid unpalatable milkweed bugs (*O*. *fasicatus*) [8] they subsequently avoid palatable, conspecific mimics [9]. Likewise, visually driven hunting spiders, such as jumping and crab spiders, have been shown to be capable of learned aversion based on colour pattern [10,11].

Given their capabilities, researchers have long postulated a role for invertebrate predators in shaping warning signals and mimicry in other invertebrates. Bates [12] himself observed aposematic butterflies (Heliconidae) in the neotropics flying freely without being persecuted by invertebrate predators including dragonflies and robberflies, despite the fact that these invertebrates were often seen attacking butterflies from other families. Shelly & Pearson [13] noted that predatory robber flies attacked chemically defended tiger beetles with orange abdomens less frequently than tiger beetles with dark abdomens and suggested that they may be partially responsible for the evolution of warning signals in this species. Work with mantids (Mantodea) shows that they can learn from experience to avoid unpalatable prey and that learning time can be shortened using more conspicuous signals such as stripes [14] or high luminance contrast [15]. These studies not only demonstrate the abilities of invertebrates to gather information on prey quality but also that they are at least theoretically capable of generating selection for unpalatable prey to evolve conspicuous signals.

Dragonflies (Odonata: Anisoptera) are major predators of Hymenoptera and Diptera [16], groups rich with models and mimics, and their behaviour and preferences could have important implications for the evolution of warning signals and mimicry in these groups. Dragonflies are highly visual predators with eyes that are specialized for detecting motion, colour (both visible and ultraviolet) and polarized light [16, 17]. Perched dragonflies are also able to discriminate the distance and trajectory to prey items and some have been shown to be highly size selective, attacking artificial prey no larger than their heads [18]. Indeed, as Bates [12] had observed, dragonflies show considerable diet selectivity in that they do not always attack the prey items that they approach [19, 20]. O’Donnell [21] for instance found that Neotropical dragonflies oriented towards wasps but rarely pursued them and suggested that this behaviour may potentially select for mimicry.

Two experimental studies conducted over a decade ago were the first to formally quantify the responses of individual dragonflies to natural and artificial prey in the field. Kauppinen & Mappes [22] evaluated the responses of the dragonfly *Aeshna grandis* to black flies and wasps, in their natural state and painted with warning colours, as well as artificial prey. All prey were presented to the dragonflies tethered on fishing line. After recording a variety of tactile and non-tactile responses of the dragonflies towards the prey, the authors suggested that prey with wasp-like coloration are avoided more by *A*. *grandis*, so such patterns may confer a selective advantage against such invertebrate predators. By contrast, in independent work presenting tethered natural and artificial prey to a range of dragonfly species, Rashed et al. [23] concluded that while dragonflies showed a preference for attacking small prey over large prey, there was no compelling evidence to support the hypothesis that conspicuous signals protect small insect prey from attack by dragonflies. Considering these two contrasting results, the question as to whether dragonflies can select for conspicuous colouration in their invertebrate prey remains unresolved.

In this paper, we have significantly expanded on previous work in two important ways. First, while Kauppinen & Mappes [22] controlled for prey size and Rashed et al. [23] contrasted attack rates on two prey sizes, here we investigated the foraging preferences of odonates for a much wider range of prey sizes. Second, we have quantified these prey size preferences for prey of two colour patterns, namely black and black-yellow striped prey. Together, this work has allowed us to investigate prey size preferences, colour pattern preferences, as well as whether prey size and colour pattern interact to provide protective benefits to prey against dragonfly predators. It is conceivable, for instance, that dragonflies preferentially avoid conspicuously marked prey but only when prey are above a certain size. This form of size-dependent interaction has been observed in caterpillars where eyespots provided protective benefits for large caterpillars but not small caterpillars [24]. Likewise, Penney at al. [25] showed that mimetic fidelity in hoverflies (Diptera: Syrphidae) increases with body size, implying that close mimicry is more important for large-bodied insects. Alternatively, warning colouration (i.e. black-yellow stripes) could provide protective benefits to smaller prey that would otherwise be absent, while dragonflies could reject larger prey regardless of colour. We therefore ask for the first time whether the attack rates of dragonflies on aerial insect prey are mediated by an interaction between prey body size and colour pattern.

One possible explanation for the contrasting results of Kauppinen & Mappes [22] and Rashed et al. [23] is that they investigated the foraging behaviour of different dragonfly species. To address this phenomenon, we first compared the foraging preferences of collections of different dragonfly species. We then directly compared the foraging preferences of two specific dragonfly species from the same family. Dragonflies are generalist and opportunistic predators, mainly feeding on aerial insect prey but they can be limited by their body size when handling and feeding since they must hold and macerate their prey [16]. Although there is some intraspecific variation in body size among dragonflies, much of the variation in size is interspecific. We therefore classified the species we tested within the community into broad size-class categories (small and large) to elucidate the role of dragonfly body size in mediating foraging preferences. Just as Olberg et al. [18] had argued, we predicted that the maximum prey size preferred by dragonflies would be relative to their body size due to handling constraints.

In a separate and more direct experiment, we compared the foraging preferences of two common co-occurring species, *Leucorrhinia intacta* (28–32 mm; head to end of abdomen) and *Libellula pulchella* (43–52 mm), in terms of their response to prey of two sizes (5 and 12 mm length). We predicted that *L*. *pulchella*, the larger of the two odonate species, would show a greater tendency to attack larger prey while *L*. *intacta* would attack only smaller prey. We also took the opportunity to test any potential sex differences in foraging, given that mature odonates are readily sexed based on colouration and/or genital organs. While the two sexes can have different nutritive requirements and time budgets (e.g. territorial defence in males, maturing ovaries in females), we did not have an *a priori* expectation of a sexual difference in foraging preference.

## Methods

We conducted two experiments: a dragonfly community prey choice experiment and a species-specific prey choice experiment. The experiments were conducted in East Field meadow (approx. 1 ha; 44°32’29” N, 76°22’17” W) near Queens University Biological Station, Ontario, Canada. All dragonflies were tested from 0900 to 1500 local time, on sunny days, with temperatures ranging from 13–30°C. Dragonflies were at particularly high density in this meadow (S1 Video) and almost certainly one of the major predators of invertebrate prey in the area. Surveys of the dragonfly community were conducted every two weeks to monitor species composition and relative abundances. These surveys were performed by first walking the perimeter of the field then spiralling inwards, towards the centre, tallying the species of individuals observed perching as we walked.

### Community prey choice experiment

**Fig 1:**
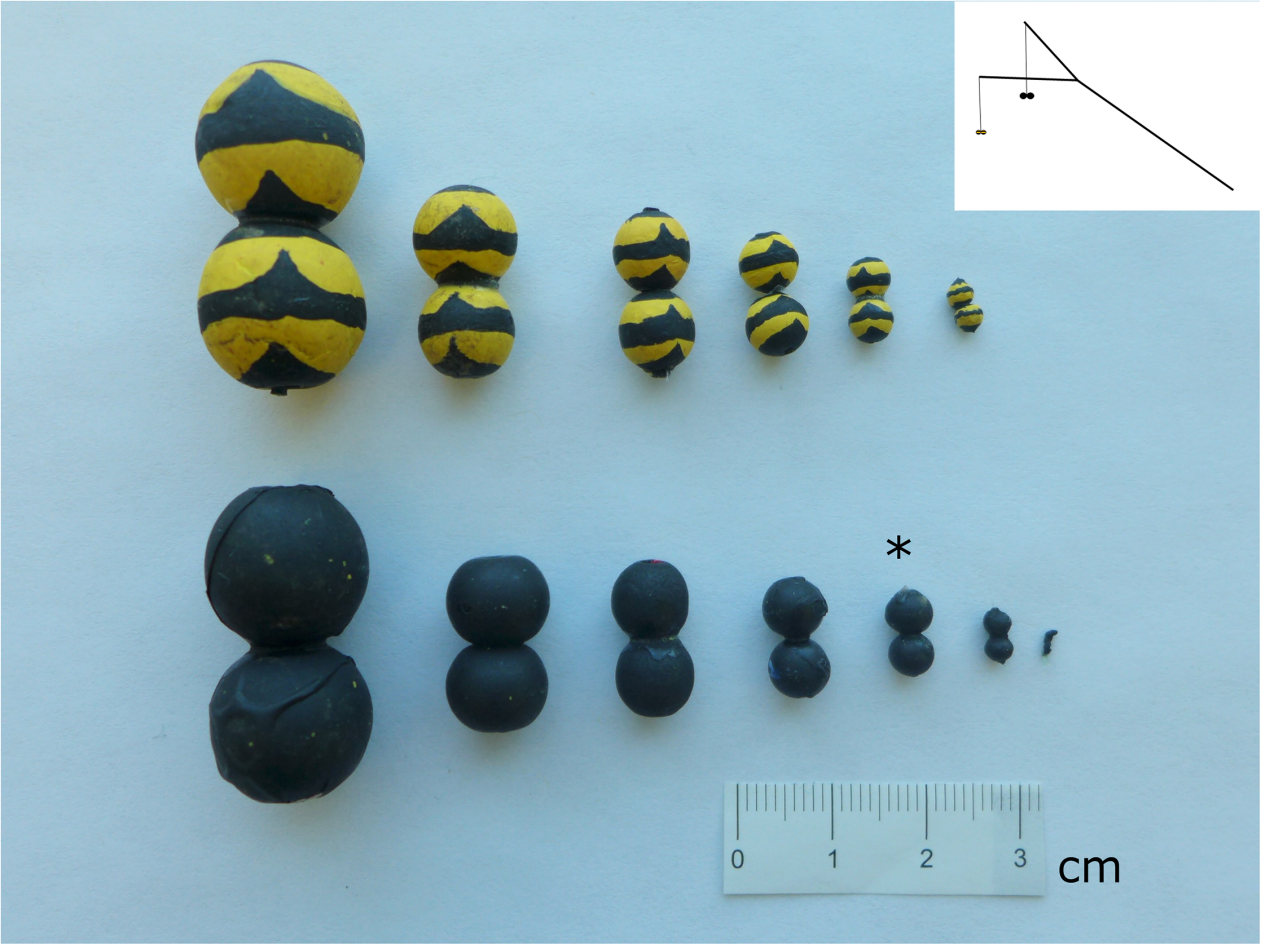
Artificial prey made from two conjoining beads, painted with acrylic paint in two colours (black and black/yellow striped) in various size treatments (2.5 (black only), 5, 9, 12, 16, 18, and 31 mm). The standard bead used in each pairwise presentation is denoted (*). Beads were offered to dragonflies using a Y-shaped stimulus rod (top right).

The community prey choice experiments were performed between 3 June—3 July and from 17 July—30 July 2015. To study the preferences of dragonflies for prey of different size and colour pattern, artificial prey were created by gluing two spherical beads of the same diameter together (loosely mimicking the shape of Hymenopteran/Dipteran prey) and painted using non-toxic acrylic paint (DecorArt Crafter’s Acrylic paint in “Bright Yellow” and “Black”) (Fig 1). Prey consisted of seven different sizes (2.5, 5, 9, 12, 16, 18 and 31 mm; lengthwise) and two colour treatments (black, and black and yellow). The black and yellow stripes were scaled such that prey of all sizes received 5 black stripes and 4 yellow stripes. However, the smallest size treatment (2.5 mm) could not be painted with stripes and was presented only in black.

The reflectance of paints was measured using the Ocean Optics USB 2000 spectrophotometer (Dunedin, Florida, U.S.A.). Spectra were measured and calibrated against a 98% white standard (Labsphere). The peak reflectance for yellow was around 570 nm, which was lower than previously recorded for vespid wasps [22, 23] while the paints were not UV reflective (S1 Fig).

Our artificial prey were presented on a pairwise 183 cm long (total length) Y-shaped rod made of bamboo (with each prong measuring 53 cm in length)–see Fig 1. Prey were hung 100 cm from the ends of each prong using dark green Power Pro microfilament braided fishing line (10 lb test; Innovative Textiles). Treatment prey were always presented pairwise with a standard bead (black 12 mm bead). To prevent potential side bias, the standard bead was alternately tethered on each prong with each presentation. This standard bead was introduced to facilitate preference standardization: the emergence times of dragonfly species did not completely overlap and with inevitable seasonal changes in environmental conditions (such as temperature and wind speed) it was appropriate to compare the number of attacks on a given prey type with the number of attacks against a constant standard model. This approach gives a relatively direct measure of the nature of preference for one phenotype over another. To present the beads, we first located a perched dragonfly and then approached it slowly while holding the rod 2–3 m above the ground. The beads were then hung approximately 0.5–1.5 m above the dragonfly and moved in a manner that mimicked natural prey movement (i.e. side to side as if flying across the field of vision). Dragonflies were given four minutes to attack prey. When a prey item was attacked (i.e. the dragonfly made direct physical contact with the bead), the type of bead (standard or treatment), size, and colour were noted. After an attack occurred, the dragonfly was caught and marked with two small dashes on the hind wing using a permanent marker, which allowed us to avoid re-testing the same dragonfly. The sex and species of all tested dragonflies were identified and recorded. The rate of re-encounter with dragonflies that had already been tested was extremely low (2 re-encounters of 1153 attempted presentations) as the density of dragonflies was extremely high. No teneral dragonflies, readily identifiable through their “glassy” wings, were tested.

In addition to our analysis of the whole community, we sought to evaluate the broad scale patterns of foraging responses of dragonfly species in relation to their body size. The foraging preferences of thirteen species of dragonflies were evaluated (Table 1). Species were classified into two (approximately equal sized) small (*Leucorrhinia intacta*, *Celithemus elisa*, *Celithemus eponina*, *Leucorrhinia*. *glacialis*, *Sympetrum spp*., *Epitheca cynosura*, *Leucorrhinia frigida*, and *Pachydiplax longipennis*) and large (*Libellula Julia*, *Libellula quadrimaculata*, *Libellula luctuosa*, *Gomphus spicatus*, and *Libellula pulchella*) size-class categories for total body length based on [26]. We tested whether there were differences in attack frequency or size preference for given prey types that could be explained by differences in the body size class of predator by fitting a generalized linear model (see below).

**Table 1:** A summary of species included in the community choice experiment analyses, their total body length (head to abdomen tip), given size-class category, and the number of individuals tested.

Species	Length (mm)	Size Category	Number of individuals tested
*Leucorrhinia intacta*	29–33	S	177
*Celithemus elisa*	24–34	S	132
*Sympetrum spp*.	24–31	S	9
*Leucorrhinia frigida*	28–32	S	1
*Pachydiplax longipennis*	28–41	S	9
*Leucorrhinia glacialis*	34–37	S	1
*Celithemus eponina*	36–42	L	94
*Epitheca cynosura*	37–44	L	2
*Libellula julia*	38–45	L	90
*Libellula quadrimaculata*	39–48	L	229
*Libellula luctuosa*	42–50	L	104
*Gomphus spicatus*	46–50	L	99
*Libellula pulchella*	52–57	L	194
Total # Individuals Tested			1141

S, small species category; L, large species category

### Species-specific choice experiment

To test for potential inter- and intra- specific (sex-based) variation in response to prey size in a more focussed way, we presented two sizes of black, artificial prey (5 and 12 mm total length) separately to two species of co-occurring dragonflies namely *Leucorrhinia intacta* and *Libellula pulchella* (29–33 and 52–57 mm lengthwise, respectively). The foraging behaviour of these species were chosen to investigate because they exhibit clear body size differences, overlap in flight times, and were abundant during the experimental period. This experiment was conducted from July 3–17 of 2015. To control for variation in weather, dragonflies were tested in temporal blocks (cycling through the two species and two prey sizes in random order). Prey were hung 100 cm down from a 183 cm long, straight bamboo pole. Unlike the pair-wise presentation in the previous experiment, these beads were offered singularly because the species co-occurred throughout the experimental period and because the 2 x 2 treatment combinations allowed us to employ a randomized block design to control for environmental variation. As such, a block effect was able to account directly for any variation in foraging behaviour that could be explained by fluctuations in environmental conditions such as temperature and wind speed.

We walked the perimeter of the field until we located one of the target species and prey were offered for a maximum of 120 seconds. We measured the latency from the presentation of the prey until either the dragonfly attacked, did not attack but stayed perched for the allotted time or flew away before the end of the allotted time. We also recorded the sex of all tested dragonflies. Given the high abundance of both species and the low re-encounter rate in the community experiment (see above), tested individuals were not marked. Once again, no teneral dragonflies were tested.

### Statistical analysis

All analyses were conducted using R v. 3. 1. 3 [27]. Given the binary nature of the response variable in our community preference tests (the dragonflies attacked either the treatment prey or standard prey) we fitted a generalized linear model assuming binomial error (i.e. a logistic regression model). This model treated prey size (as a continuous variable), colour (categorical), and their interaction, as predictors. To compare the attack rates (proportion of attack on treatment bead) of different dragonfly size classes (two levels) we fitted a second logistic regression model that included dragonfly size class as a fixed factor (since there were only two levels), with all but the highest order (3 way) interaction. This enabled us to elucidate how the probability of a dragonfly attacking the treatment bead might vary with prey size (continuous), prey colour (categorical), and dragonfly size (categorical) as well as their component two-way interaction.

For the species-specific prey choice experiment, we fitted a Cox proportional hazards model using coxph function from the R survival package to investigate whether dragonfly species, dragonfly sex, temporal block and prey size influence the time to attacking prey in our species-specific experiments. Three distinct endpoints were recognized in this survival analysis. First, if the dragonfly attacked the bead, we noted its latency to attack in seconds. Second, if the dragonfly flew away before the allotted time, the time at which observations ceased was recorded (a censored data point). Finally, if the dragonflies did not attack the offered bead after the 120 seconds and stayed perched, then the event was considered right censored. Our time to event analysis considered dragonfly species, dragonfly sex, bead size, and each of their two-way interactions as categorical predictors.

## Results

### Community prey choice experiment

**Fig 2:**
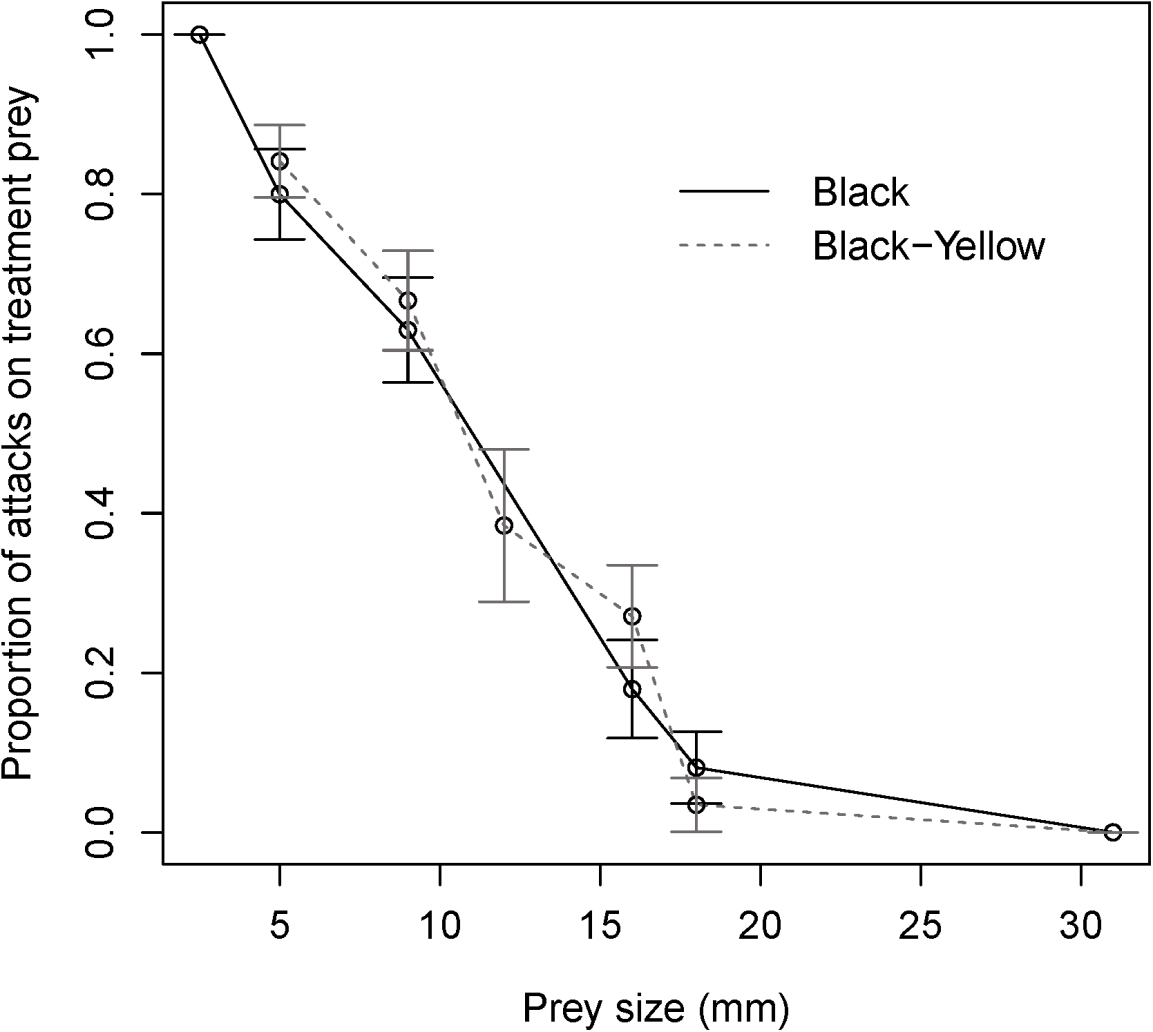
The proportion of attacks on treatment beads (compared to standard bead) by a community of adult dragonflies on prey of varying size (2.5 (black only), 5, 9, 12, 16, 18, and 31 mm) in two colour patterns (black and black/yellow striped). Error bars show the binomial 95% confidence intervals.

A total of 1153 dragonflies were presented with a pairwise choice. 46% of the dragonflies tested made an attack and of the prey attacked, overall 48% and 52% attacked the standard and treatment bead, respectively. Prey size was a strong determinant as to whether dragonflies attacked the treatment bead, since the smaller the treatment prey, the more likely they were to be attacked (z = 8.698, P < 0.001). However, there was no effect of prey colour pattern on dragonflies’ attack decisions (z = 0.585, P = 0.559) and no evidence for an interaction (z = -0.785, P = 0.433). A sequential analysis of deviance based on log likelihood ratio tests yielded analogous results. Thus, the probability of a treatment bead being attacked by dragonflies increased as prey size decreased (Fig 2), regardless of colour pattern.

**Fig 3:**
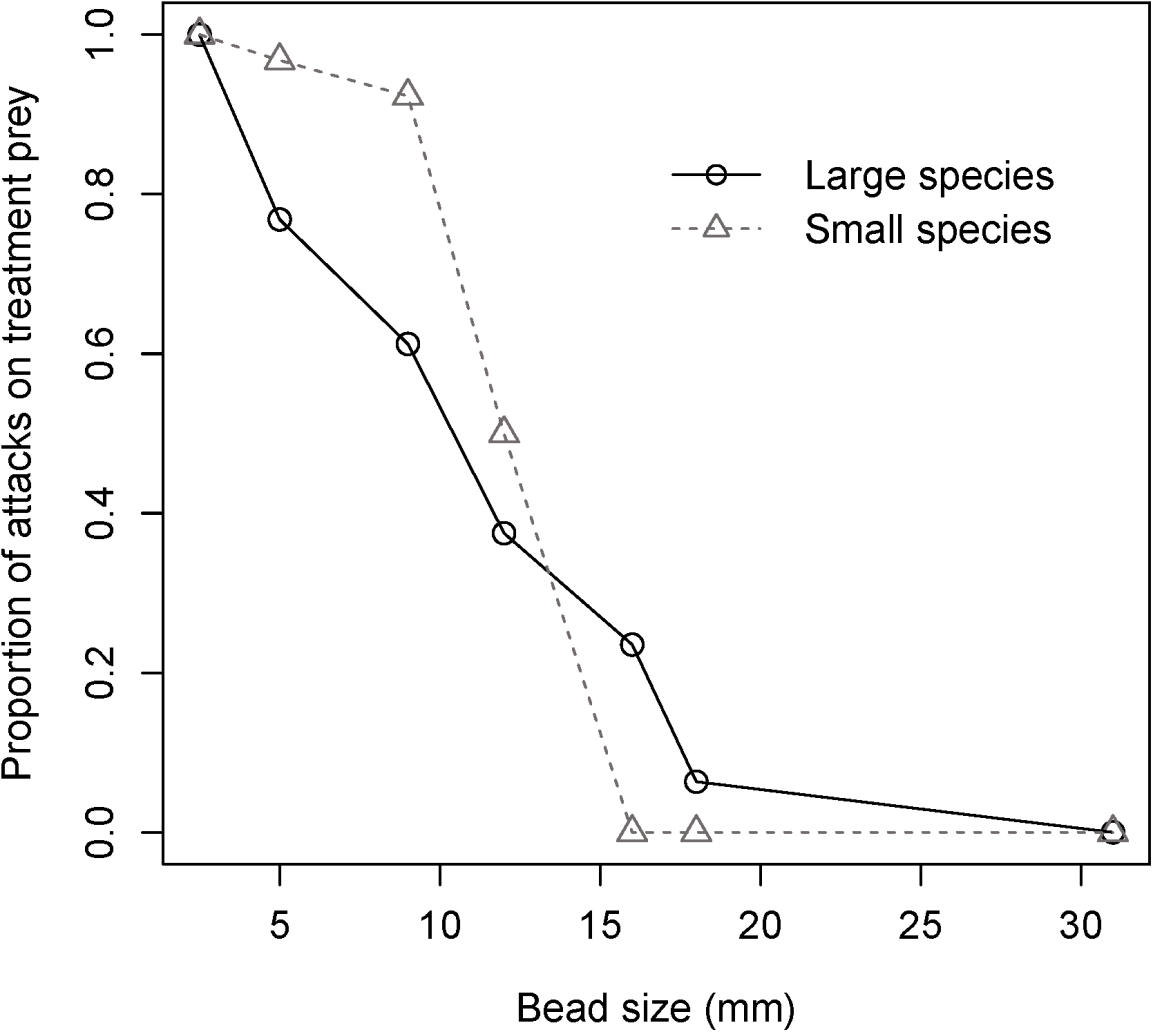
The probability of attack on treatment bead (compared to standard bead) by smaller dragonflies (*C*. *elisa*, *C*. *eponina*, *L*. *intacta*, *L*. *glacialis*, *Sympetrum spp*., *E*. *cynosura*, *L*. *frigida*, *P*. *longipennis*) compared to larger dragonflies (*L*. *quadrimaculata*, *G*. *spicatus*, *L*. *pulchella*, *L*. *julia*, and *L*. *luctuosa*) on prey of varying size (2.5 (black only), 5, 9, 12, 16, 18, and 31 mm) for both colour pattern treatments.

When evaluating the attack rates of dragonflies of different size classes on the standard bead compared to beads of different size classes, prey size (z = 4.322, P < 0.001) and dragonfly size class (z = 2.340, P < 0.0193) significantly affected the response of dragonflies such that smaller dragonflies only attacked prey up to 12 mm but larger dragonflies attacked across the range of prey sizes (Fig 3). Once again, prey colour pattern was not significant (z = 0.086, P = 0.9331). The interaction of prey colour pattern with prey size and dragonfly size class were not found to be significant (colour x prey size, z = 0.457, P = 0.6474; colour x dragonfly size, z = -0.349, P = 0.7269). Likewise, the interaction between prey size and dragonfly size class was not significant, although marginal (z = -1.731, P = 0.0834). A sequential analysis of deviance generated analogous results, with the exception that the prey size x dragonfly size interaction was significant (χ^2^_1_ = 6.039, P = 0.0134).

### Species-specific choice experiment

**Fig 4:**
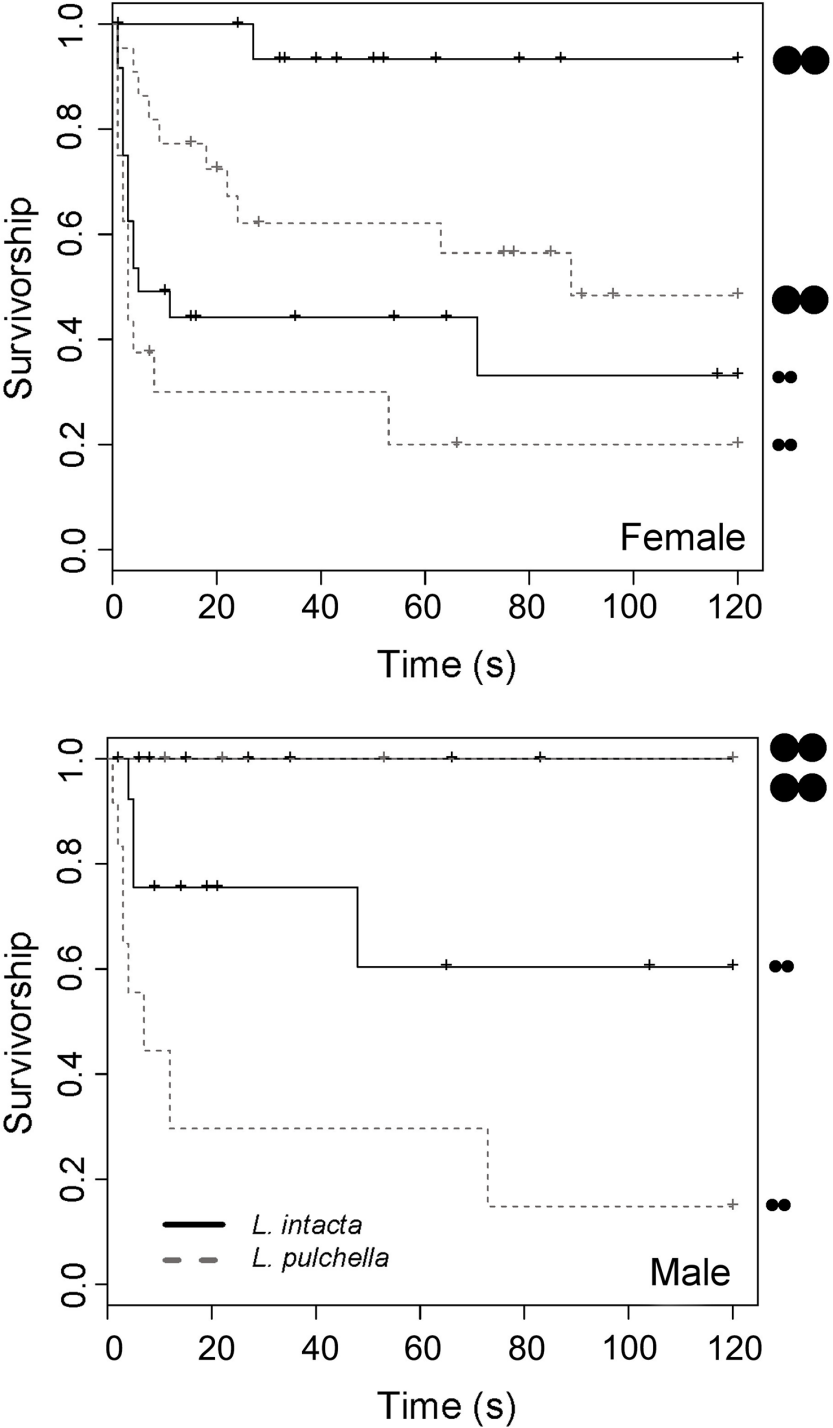
Cumulative survivorship curves for prey (5 and 12 mm; lengthwise [depicted diagrammatically]) against female (top) and male (bottom) dragonflies of two species (*L*. *intacta and L*. *pulchella*). Censored observations (i.e. when dragonfly flew away without attacking) are denoted by “+”.

A total of 49 attacks from 131 presentations were made by the two species of dragonfly. 76% of attacks were made by females, of both species, and but 60% of all presentations were to females (Table 2). The probability of prey being attacked was higher overall when prey were presented to *L*. *pulchella* than *L*. *intacta* (z = -2.947, P = 0.0032), as *L*. *pulchella* attacked both prey sizes while *L*. *intacta* mainly attacked 5 mm prey (Fig 4). Attack rates on prey were also affected by prey size (z = 4.209, P < 0.001) and sex of dragonfly (z = 2.355, P = 0.0185). Unlike the community experiment, the interaction between dragonfly species and the size of prey on time to attack was not significant (z = 1.539, P = 0. 1238), and neither was the sex x prey size interaction (z = 0.697, P = 0.4857). A sequential analysis of deviance yielded analogous results.

**Table 2:** Number of prey presentations and attacks made by female and male *L*. *intacta* and *L*. *pulchella*.

Species	Female		Male	
	Offered	Attacked	Offered	Attacked
*L*. *intacta*	41	15	34	4
*L*. *pulchella*	38	22	18	8

Offered, artificial prey of either 5 or 12 mm were presented and dragonflies were allotted 120 s to attack; Attacked, the dragonfly made physical contact with the offered artificial prey.

## Discussion

The aim of this study was to investigate the foraging choices of dragonflies in relation to the colour and size of presented prey. Overall, dragonflies preferred smaller prey. However, smaller dragonflies tended to attack prey up to 12 mm in length while larger dragonflies attacked across the range of prey sizes presented. The species-specific experiment further supported these results, as *L*. *intacta*, the smaller species of the two tested, attacked primarily small prey. In addition, female dragonflies of the two species were more likely than males to attack any form of prey. Together, the results suggest that smaller prey are strongly preferred by this community of dragonflies but that they exhibit no preference for black over black-yellow prey of any given size.

Our conclusion that prey colour pattern was not a key feature influencing dragonfly attacks is consistent with the results of Rashed et al. [23]. With the expansion of this design to cover a range of prey size treatments in two different colour patterns, we were also able to rule out a potential interaction between prey colour pattern and size. Colour cues are clearly used in conspecific identification and communication, since odonates exhibit a wide variety of colours and patterns that can vary intra- and inter-specifically [28, 29]. “Percher” dragonflies have specialized ommatidia on the dorsal portion of their eye which allows them to see prey against a bright sky backdrop [30]. While every attempt was made to present prey in a natural setting in this field experiment, with prey viewed against a backdrop of vegetation as well as the sky, it is conceivable that colour detection was difficult in those instances where illumination was from directly behind the prey.

In contrast to coloration, dragonflies were clearly highly size selective, with an overall tendency of dragonflies to prefer smaller over larger prey. Indeed, we could find no lower limit on body size, with dragonflies preferring our very smallest prey most strongly. Our results also provide a plausible explanation for the phenomenon, in that predator body size was also found to be a strong determinant of prey size preferences. Collectively, these results suggest that handling constraints and/or gape limitations [31, 32] influence foraging decisions, so that large prey are simply too unwieldy to be profitable. Many invertebrate raptorial feeders are known to have preferences for specific prey sizes [33, 34]. Our results are especially interesting considering the turnover of species that occurs in this area. Based on observations of dragonfly community species composition during the experiment, medium-large species were more prominent early in the season while smaller species were dominant later in the season. Thus, changes in dragonfly species composition could also mean subtle changes in the nature of selection pressure on prey sizes.

In our species-specific choice experiment, females attacked the presented prey more readily than males (47% vs 23%, of total presentations made) and females tended to attack larger prey. To date, very little work has been done on sex-based foraging preferences in dragonflies. It is possible that the differences we observed in our experiment are due to differences in reproductive demands. For example, during and around the time of reproduction in adult dragonflies, males and females can differ in the primary energy allocations that are dictated by their relative reproductive roles. Prior to sexual maturity in adults, weight gain and energy assimilation in females is focused on ovarian maturation and thoracic flight muscles (to search for suitable oviposition sites) whereas males solely dedicate their weight gain to thoracic flight muscles, likely necessary for territoriality, mate searching, and/or mate guarding [16]. In turn, these differences can alter foraging behaviour with females showing higher foraging rates, and a higher willingness to attack larger prey. Although we found no evidence of a significant interactive effect in the species we investigated, sex-based differences can also vary between species; in pre-reproductive stages, males of *Pachydiplax longipennis* and *L*. *pulchella* in enclosures gained more weight than females [35, 36] but in an analysis of 54 species (8 families), Anholt et al. [37] found that on average males and females gained 84% and 125% respectively, of their original body weight during maturation. In our study, it is likely that we tested a range of dragonflies from varying adult life stages–pre- reproductive (falls between teneral stage and the reproductive stage, which often entails dragonflies feeding at higher rates to prepare for reproductive processes), reproductive, and post-reproductive–and it would be difficult to test the effect of life stage on prey preference using our data. However, our results give a coarse indication of how the sex of two species of different sized dragonflies can influence foraging preference.

Our study indicates that the dragonflies in our community study selectively forage for smaller prey but do not differentiate between black and black-yellow prey, regardless of their size. In testing a preference for differently patterned prey across a broader range of prey sizes, this study extends previous work on dragonfly foraging in the context of warning colouration [22, 23] and provides further insight on the foraging preferences and behaviour of a prominent aerial invertebrate predator. We conclude that dragonflies are unlikely to play a significant role in selecting for visual warning signals but an increase in size provides protective benefits to the prey, to dragonfly predators at least. Note however that dragonflies may impose selection on other aspects of the prey such as distastefulness, aggressive behaviour, morphological protection (e.g. hardened, leathery cuticle) that are all experienced after capture. Indeed, we occasionally witnessed dragonflies catching natural prey but dropping them after sampling, indicating the potential for taste-rejection behaviour. Likewise, in preliminary hand-feeding experiments, dragonflies ultimately rejected Hymenoptera (e.g. sweat bees, wasps, etc.) that were offered unless they could break the cuticle. It has long been suggested that warning signals can sometimes promote more cautious (“go-slow”) sampling behaviour following capture [38]. An obvious next step for this work is therefore to monitor the survival of natural prey and evaluate the factors–including coloration–that influence the survival of prey types following capture.

## Supporting information

S1 Fig Spectral curves for the black and yellow paint used to create artificial prey. (TIFF)

S1 Video A video (16 sec) demonstrating the abundance of foraging dragonflies on a sunny day within the period of experimentation. (MP4)

S1 Dataset Field data used for the analysis of community prey choice including the date of presentation, dragonfly species, dragonfly sex, dragonfly size class, treatment bead colour, treatment bead size, treatment bead placement on Y-prong, attack (1) or no-attack (0), attack on treatment or control bead, time to attack, time of attack, temperature at time of attack, and wind speed at time of attack. (CSV)

S2 Dataset Field data used for the analysis of prey survival in the presence of *Leucorrhinia intacta* and *Libellula pulchella* including date of presentation, dragonfly species, dragonfly sex, bead size, attack (1) or no-attack (0), and the time (sec) to attack. (CSV)
