# Supplementary figures and images for: Response of adult dragonflies to artificial prey of different size and colour
Source: PLoS One. 2017 Jun 29;12(6):e0179483. doi: 10.1371/journal.pone.0179483 (PMC5491015; doi:10.1371/journal.pone.0179483)

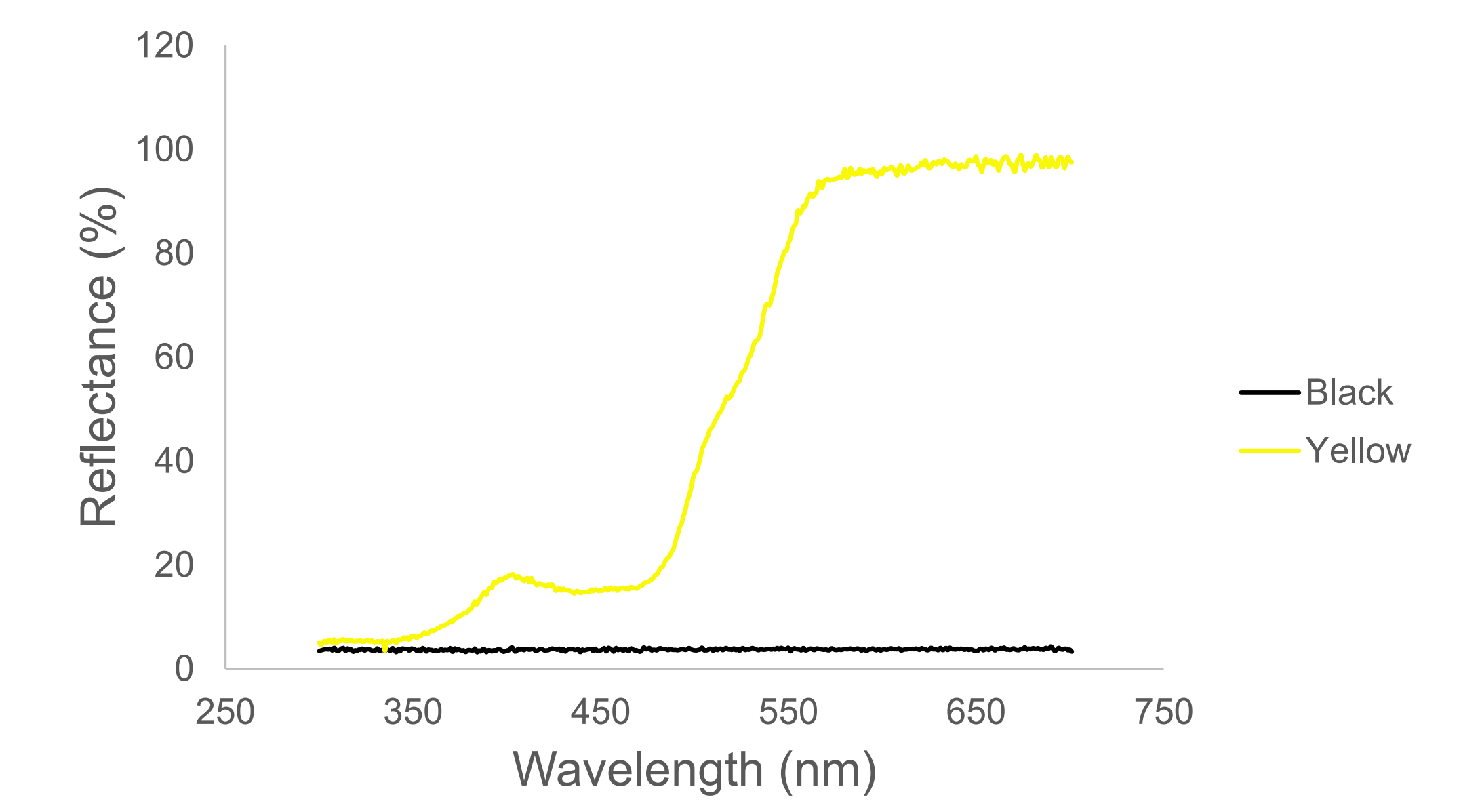

Supplement: S1 Fig — (TIFF) [file pone.0179483.s001.tiff]
